# Subglottic Stenosis After Endonasal Resection of Juvenile Nasopharyngeal Angiofibroma

**DOI:** 10.7759/cureus.43922

**Published:** 2023-08-22

**Authors:** Virali D Shah, Jordon G Grube, Lara Reichert

**Affiliations:** 1 Otolaryngology - Head and Neck Surgery, Albany Medical College, Albany, USA; 2 Otolaryngology - Head and Neck Surgery, Albany Medical Center, Albany, USA

**Keywords:** pediatrics emergency, otolaryngology case report, endonasal endoscopic surgery, skull base surgery, airway stenosis, dyspnea, pediatric otolaryngology, endoscopic surgery, juvenile nasopharyngeal angiofibroma, subglottic stenosis

## Abstract

Subglottic stenosis (SGS), the narrowing of the upper trachea, can be an acquired condition in pediatric patients. Presenting with varying degrees of dyspnea and stridor, acquired SGS is most commonly due to intubation. Airway stenosis is often not considered a surgical complication, and no literature on acquired SGS after endoscopic sinus surgery exists. We present a unique case of a 13-year-old male with juvenile nasopharyngeal angiofibroma (JNA), who developed SGS in the setting of progressive dyspnea six weeks after endonasal resection of his mass. He required urgent intubation prior to preoperative embolization and endonasal surgery, which prolonged his total intubation period. After the patient was found to have acquired SGS, he eventually required serial dilation to treat his stenosis. The presentation and operative course of this patient, along with images and pathologic findings, are discussed. Based on an extensive literature review of PubMed, Medline, and Google Scholar, there have been no cases discussing SGS development post-intubation after endonasal surgery or in association with JNA. Acquired SGS can present as a life-threatening airway obstruction in pediatric patients. With the rise of endoscopic skull base surgery and the prevalence of JNA, this case study sheds light on the detection and management of SGS post-operatively.

## Introduction

Subglottic stenosis (SGS), the narrowing of the upper airway below the vocal folds, can be an idiopathic, congenital, or acquired complication in pediatric patients [1]. Classic symptoms of SGS include varying degrees of dyspnea and stridor [2]. Idiopathic SGS is a rare, fibrotic disease with an unknown pathogenesis that can be mistaken for new-onset asthma [3]. Small cricoid cartilage sizes or other laryngeal abnormalities can cause respiratory difficulty at birth in those with congenital SGS [4]. However, the most common cause of SGS is acquired from prolonged intubation [2]. The estimated incidence of acquired SGS after prolonged endotracheal intubation is about 11% [5]. The use of less sedation, longer duration of intubation, endotracheal tube size, and co-existing acid reflux are significant risk factors for acquired SGS development post-intubation [2,6,7]. SGS secondary to endotracheal intubation has been well-researched. While many surgical procedures require endotracheal intubation to protect the airway and ventilate the patient, the risk of airway damage is not often considered among possible surgical complications. Airway stenosis after surgery is not well studied, and there is no existing literature on acquired SGS after endoscopic sinus surgery.

## Case presentation

History and examination

A 13-year-old male presented to the otolaryngology clinic for evaluation of progressive dyspnea six weeks after resection of his juvenile nasopharyngeal angiofibroma (JNA) with endoscopic skull base surgery. The patient originally presented to the emergency department with a one-week history of nasal congestion, mild epistaxis, and decreased visual acuity. Ophthalmology was consulted to assess his decreased visual acuity, and the patient underwent CT and MRI. CT and MRI identified a large skull base mass within his pterygopalatine fossa with extension into the infratemporal fossa, cavernous sinus, middle fossa, nasopharynx, and bilateral nasal cavities. The imaging was consistent with a JNA. The patient underwent preoperative embolization with neuro-interventionalists, but his hospital course was complicated by urgent intubation. The patient had recurrent apneic episodes with frequent desaturation in the setting of noted upper airway obstruction. The anesthesia staff performed an uncomplicated, yet urgent, overnight intubation with a 7.0-mm endotracheal tube, which was successful after the first attempt. Once the patient was intubated and preoperative embolization was completed, the decision was made to proceed with endoscopic skull base surgery to resect the tumor.

Operative procedure

Under general anesthesia, the patient underwent a combined endoscopic and endoscopic assisted open sublabial approach to the right infratemporal fossa, pterygopalatine fossa, and middle cranial fossa with a right Caldwell-Luc procedure. The surgery consisted of a right-modified medial left maxillectomy, bilateral total ethmoidectomy, bilateral sphenoidotomy, posterior septectomy, and bilateral middle turbinate reduction. The tumor was resected from the ventral skull base, and the middle cranial fossa was subsequently repaired without complications. The total duration of surgery was 4.5 hours. 

Histopathologic analysis was completed to confirm the diagnosis of the tumor. The tumor exhibited focal intra-tumoral organizing hemorrhage, with epithelial surface ulceration and erosion. Immunohistochemistry stains showed that the tumor was positive for beta-catenin and androgen receptors, with focal staining for CD34 and negative results for STAT-6. These histologic findings confirmed the diagnosis of JNA. 

Postoperative course

The patient recovered from surgery without any complications and had no concerns during his post-operative hospital stay. He stayed intubated for 20 hours after surgery and was successfully extubated the following morning. In the postoperative period, the patient denied any nasal congestion or epistaxis, and his visual symptoms had also resolved. One week after surgery, the patient had a follow-up appointment, during which he reported some difficulty with nasal breathing and some brown-bloody nasal discharge. He underwent bilateral rigid nasal endoscopy with debridement to remove any crusting, necrotic tissue. This symptomatically improved his nasal breathing. The patient reported doing well at his two-week follow-up visit.

**Figure 1:**
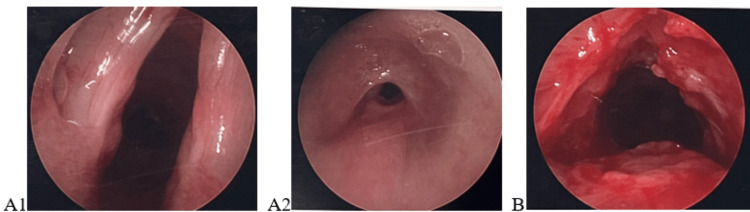
Rigid bronchoscopy photos demonstrating stenosis before (A1, A2) and after dilation (B).

However, six weeks following surgery, the patient presented to the otolaryngology clinic with difficulty breathing. He was accompanied by his brother who observed the patient having difficulty breathing in school for two weeks. The patient had momentary cessations in breathing approximately three times per day while sitting in class. In addition, the patient’s exercise tolerance had significantly declined, and he had stridorous breathing at baseline. On physical exam, the patient was found to have loud biphasic stridor at rest, with no lesions or abnormalities on anterior rhinoscopy. The evaluation was followed by a bilateral rigid nasal endoscopy, which showed well-healing sinuses from surgery and no residual tumor. Flexible laryngoscopy visualized significant narrowing below the vocal cords consistent with SGS. The patient underwent urgent operative assessment and intervention. Intraoperative bronchoscopy showed grade 3 SGS, according to the Cotton-Meyer classification system, extending into the cervical trachea with a 3-cm segment of stenosis. The stenosis was serially dilated resulting in a grade 1 SGS (Figures 1A, 1B).

**Figure 2:**
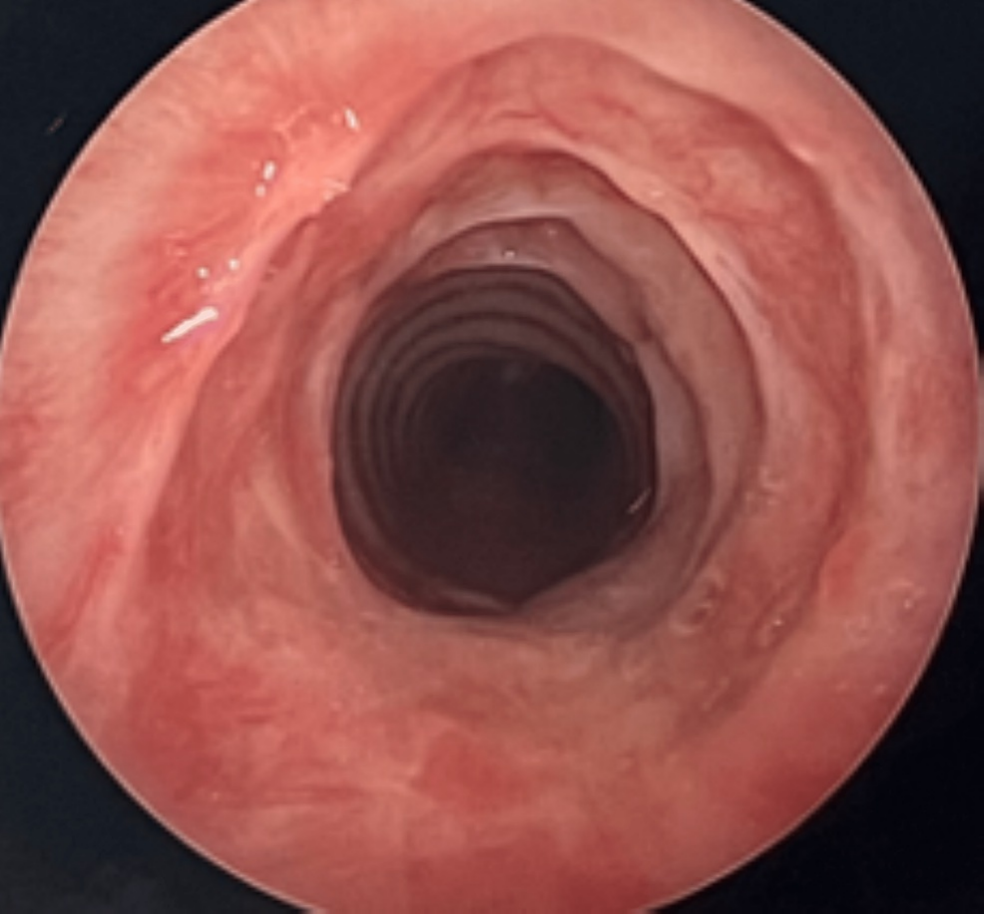
Rigid bronchoscopy results two weeks after the initial dilation procedure

Triamcinolone was injected circumferentially into the stenosed region. The patient had a repeated balloon dilation and injection two weeks later (Figure 2). At the one-month, three-month, and six-month follow-up appointments, the patient was doing well. His biphasic stridor resolved, and he had no dyspnea or exercise intolerance.

## Discussion

Acquired SGS is theorized to result from trauma that leads to scarring and granulation of tissue over time. It can present as a life-threatening airway obstruction in pediatric patients, warranting a high degree of clinical suspicion [8]. Over 90% of trauma leading to SGS is from prolonged endotracheal intubation [9]. However, external trauma from penetrating, blunt, and strangulation injuries to the oropharyngeal region can also result in acquired SGS [10]. Other causes of acquired SGS in the pediatric population include severe laryngopharyngeal acid reflux, infection, and tracheotomy [11].

The role of surgery in the development of SGS is not well known. Three studies have found an association between SGS and cardiac surgery [12-14]. However, no studies or case reports discuss the development of SGS after endoscopic skull base surgery or other otolaryngologic surgeries. Endoscopic skull base surgery has been increasingly performed in the pediatric population, due to high rates of success and long-term efficacy [15]. While endonasal surgeries typically have shorter duration times when compared to non-endoscopic surgeries, it is important to consider other factors that may extend the intubation period for patients undergoing endonasal surgery. For instance, JNAs are frequently resected by endonasal surgery. These tumors often require multiple sedation procedures for workup and treatment, including sedated MRI, angiography and embolization, and surgical resection. In addition, certain patients, such as our pediatric JNA patient, may be at higher risk for preoperative upper airway obstruction requiring earlier intubation prior to endonasal surgery. Therefore, it is crucial to understand not only the potential airway-related complications from this common surgical approach but also the treatment options available for post-surgical airway stenosis.

Treatment for acquired SGS has improved with the development of endoscopic procedures. Mild to moderate stenosis can be treated successfully with balloon dilation, as done in this patient [16]. Other endoscopic options for treatment include a cricoid split with or without graft placement. Severe SGS frequently requires tracheostomy. Laryngotracheal reconstruction or cricotracheal resection are other surgical options in severe or refractory cases [17].

Our patient’s case is unique because it is the first-ever reported case of acquired SGS after endonasal surgery. It sheds light on the possibility of developing SGS after endonasal surgery when preoperative intubation and embolization were required. While endotracheal intubation, which is a known risk factor for airway stenosis, was required for this patient's embolization and subsequent endonasal resection, the degree of resultant stenosis was unusual, especially considering that muscle relaxant was used during both anesthetic periods. Nevertheless, it is possible that this patient's necessity for preoperative embolization a day prior to surgery was a potential source for prolonged intubation. Another factor to consider was the duration of intubation after the surgery was completed. Our patient remained intubated for about 20 hours after the tumor was resected. While extubation following the surgery was considered, the anesthesia team decided to defer extubation immediately following surgery and elected to extubate him the following day. It is also unclear whether JNA patients have increased inflammatory tendencies, which may increase their baseline risk for tracheal mucosal edema and subsequent stenosis following airway instrumentation. This patient did require fairly emergent intubation due to sudden obstruction from the nasopharyngeal component of his mass, so future discussions with pediatric JNA patients and their families should include concerns for airway obstruction prior to mass resection. Furthermore, this patient’s symptoms of dyspnea and stridor resolved after two successful endoscopic procedures despite a high-grade stenosis at presentation. While the existing literature discusses how to treat SGS in pediatric patients and the role of extended-duration intubation in pathogenesis, few studies highlight the risk of intubation for elective procedures or sedated imaging in developing stenosis.

## Conclusions

This is the first report of acquired SGS after airway instrumentation for endoscopic skull base surgery in a pediatric patient. Studies have shown high rates of success with endoscopic skull base surgery in pediatrics. Endotracheal intubation, which is required for endonasal resection, is a known risk factor for airway stenosis. It is important to consider ways to minimize the duration of intubation for pediatric patients undergoing endonasal surgery, especially for JNA resection which may require multiple sedative procedures. It is also unclear whether pediatric JNA patients may have increased inflammatory tendencies making them more susceptible to tracheal edema and airway stenosis after intubation. Regardless, surgeons should maintain a high degree of clinical suspicion in patients presenting with dyspnea and stridor weeks to months post-operatively. Endoscopic procedures, such as balloon dilation, continue to be effective treatment options for SGS.

## References

[1] Jagpal N, Shabbir N (2021). Subglottic Stenosis. https://www.ncbi.nlm.nih.gov/books/NBK563265/.

[2] Ho AM, Mizubuti GB, Dion JM, Beyea JA (2020). Paediatric postintubation subglottic stenosis. Arch Dis Child.

[3] Aravena C, Almeida FA, Mukhopadhyay S, Ghosh S, Lorenz RR, Murthy SC, Mehta AC (2020). Idiopathic subglottic stenosis: a review. J Thorac Dis.

[4] Schroeder JW Jr, Holinger LD (2008). Congenital laryngeal stenosis. Otolaryngol Clin North Am.

[5] Schweiger C, Marostica PJ, Smith MM, Manica D, Carvalho PR, Kuhl G (2013). Incidence of post-intubation subglottic stenosis in children: prospective study. J Laryngol Otol.

[6] Arianpour K, Forman SN, Karabon P, Thottam PJ (2019). Pediatric acquired subglottic stenosis: associated costs and comorbidities of 7,981 hospitalizations. Int J Pediatr Otorhinolaryngol.

[7] Schweiger C, Manica D, Pereira DR (2017). Undersedation is a risk factor for the development of subglottic stenosis in intubated children. J Pediatr (Rio J).

[8] Ghosh A, Leahy KP, Singhal S, Einhorn E, Howlett P, Cohen NA, Mirza N (2016). A murine model of subglottic granulation. J Laryngol Otol.

[9] Cuestas G, Rodríguez V, Doormann F, Bellia Munzón P, Bellia Munzón G (2018). Endoscopic treatment of acquired subglottic stenosis in children: predictors of success. Arch Argent Pediatr.

[10] Pookamala S, Thakar A, Puri K, Singh P, Kumar R, Sharma SC (2014). Acquired subglottic stenosis: aetiological profile and treatment results. J Laryngol Otol.

[11] Fligny I, François M, Aigrain Y, Polonovski JM, Contencin P, Narcy P (1989). Subglottic stenosis and gastroesophageal reflux (Article in French). Ann Otolaryngol Chir Cervicofac.

[12] Cote CL, Melong J, Tremblay P (2021). Long-term laryngotracheal complications following cardiac surgery. J Card Surg.

[13] Caruso G, Van Deenen D, Willems A, Van Der Linden P (2019). Laryngotracheal stenosis in children following cardiac surgery: a retrospective review. Eur J Anaesthesiol.

[14] Kruse KE, Purohit PJ, Cadman CR, Su F, Aghaeepour N, Hammer GB (2017). Subglottic stenosis following cardiac surgery with cardiopulmonary bypass in infants and children. Pediatr Crit Care Med.

[15] Kahilogullari G, Meco C, Beton S (2020). Endoscopic transnasal skull base surgery in pediatric patients. J Neurol Surg B Skull Base.

[16] Marston AP, White DR (2018). Subglottic stenosis. Clin Perinatol.

[17] Monnier P (2018). Partial cricotracheal resection and extended cricotracheal resection for pediatric laryngotracheal stenosis. Thorac Surg Clin.

